# The Prolo Scale: history, evolution and psychometric properties

**DOI:** 10.1007/s10195-013-0243-1

**Published:** 2013-05-10

**Authors:** Carla Vanti, Donatella Prosperi, Marco Boschi

**Affiliations:** 1 University of Bologna, Bologna, Italy; 2 University of Padova, Padova, Italy; 3 Via Gramsci 19, 40012 Calderara di Reno, Bologna Italy; 4 University of Genova, Genova, Italy; 5 University of Trieste, Trieste, Italy

**Keywords:** Outcome assessment, Questionnaires, Orthopedic surgery, Spinal fusion, Low back pain

## Abstract

**Background:**

The Prolo Scale (PS) is a widely accepted assessment tool for lumbar spinal surgery results. Nevertheless, in the literature there is a dearth of consensus about its application, interpretation and accuracy. The purpose of this review is to investigate the evolution of the PS from its introduction in 1986 to the present, including an analysis of different versions of the scale and research on the existing studies investigating its psychometric properties.

**Materials and methods:**

PubMed, Cochrane Library and PEDro databases were searched. Studies in English, Italian, French, Spanish and German published from 1986 to December 2012 were analyzed.

**Results:**

The original lumbar surgery outcome scale consisted of two Likert-type scales (economic and functional). There are three more versions of the scale: Schnee proposed one consisting of 10 items, Brantigan made one with 20 items and introduced 2 more subscales (pain and medication), and Davis adapted the scale for the cervical spine. PS is often mentioned without any specific reference to the version used; therefore, a homogeneous comparison of studies is difficult to achieve. Several authors agree on the need to embrace a multidimensional measuring system to evaluate low back pain (LBP), but there is still no consensus regarding the most reliable tool. To date, PS has been mostly used as secondary outcome measure in association with validated primary measures for LBP.

**Conclusions:**

The Prolo Scale has been adopted for clinical examination for 20 years because it is easy to administer and useful to compare significant amounts of data from surgical studies carried out at different times. Although several authors demonstrated the scale sensitivity among a battery of tests, no thorough validation study was found in the current literature.

## Introduction

Current literature stresses the relevance of adopting outcome measures to assess the effectiveness of conservative or surgical treatments. Among different evaluation tools, questionnaires are widely employed for their simplicity, reproducibility and acceptability.

The patients’ opinion about treatment results is recognized as a relevant part of the assessment of surgical procedures. In 1986, Donald J. Prolo and colleagues [1] developed the Prolo Scale (PS), with the aim to introduce a widely accepted tool to evaluate the results of lumbar spine surgery.

This scale is easy to administer, semi-quantitative and independent from the surgical technique. It provides an index of surgical efficacy and is useful to compare studies carried out at different times and on heterogeneous patient populations. To date, this scale has been used either as a primary outcome or in association with other outcome scales, and it is known as the Prolo Scale, Prolo score, Prolo Economic Functional Rating Scale, anatomic economic functional grading system or other “modified” Prolo Scale.

Several modifications concerning the name and structure of this scale (e.g., item type, item number, anatomical district of interest) were observed in the literature. Moreover, the cutoff for clinical success was commonly rated as excellent, good, fair or poor, but some specifications for each item according to the criteria of Odom [2] and MacNab [3] were recognized. Although several authors employed the PS, no literature review analyzed the characteristics and accuracy of this questionnaire.

This study aimed at investigating the evolution of the PS from its introduction to the present, including the analysis of different versions of the scale, the assessment of its psychometric properties and research on non-English validated versions.

## Materials and methods

The research was carried out by consulting the PubMed, Cochrane Library and PEDro databases.

This research strategy was applied: (Prolo score OR Prolo Scale) AND (outcome assessment OR outcome measure OR clinical success) AND (lumbar surgery OR lumbar fusion OR spinal surgery).

Further research was performed using the following keywords: valid* outcome assessment, economic and functional outcome, low back pain (LBP), sciatica, disc herniation, spondylolisthesis and stenosis.

We collected only studies on humans in English, Italian, French, Spanish or German and published from 1986 to December 2012.

Two independent researchers (CV, DP) identified and selected the studies and processed data with the same method. A third reviewer (MB) was consulted in case of disagreement.

Results were organized into different sections: description, origin, diffusion, modified versions and psychometric properties.

## Results

**Fig. 1:**
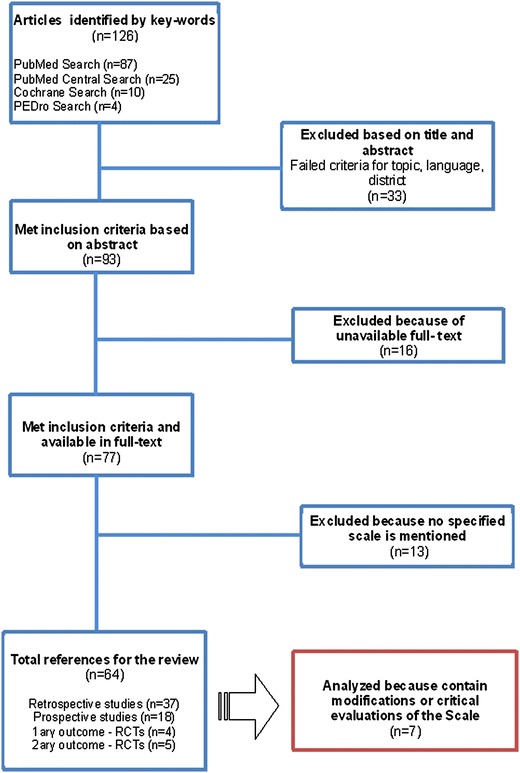
Flow chart

Initially, 126 studies were identified. Afterward, 33 were excluded because they did not match the inclusion criteria, 16 were excluded because no full text was available, and 13 were excluded because they did not mention the adopted version. Hence, the review was conducted on 64 studies (Fig. 1), out of which 7 not only administered the scale, but also analyzed it and considered the factors that influenced its accuracy (Table 1).

**Table 1:** Table of selected articles

Article	Type of study	Patient sample/follow-up	Aim of study	Comments
Berger [10]	Retrospective	1,000 workmen’s compensation patients/mean follow-up 51 months	Clinical outcome assessment measured on independent neurological and orthopedic examination vs disability score (PS)^a^	Influence of psychosocial factors and chronic pain. Sample selection bias?
Blount [42]	Review	Revision of 27 studies on spinal fusion published from 1990 to 2000	Reporting the most validated outcome measures and proposing a multi-dimensional set for spinal fusion outcome	Prolo economic score (Schnee) is recommended for return-to-work assessment. Prolo functional score is not recommended for disability assessment
Brantigan [51]	Prospective	221 patients treated with PLIF^b^ and pedicle screw fixation (I/F cage)/2 years follow-up	Testing the safety and efficacy of an interbody fusion device	Different version of PS (20 items instead of 10)
Porchet [11]	Cohort Study	394 consecutive patients with sciatica/1 year follow-up	Association between clinical examination (PS, VAS^c^, RMDQ^d^, SF-36^e^) and radiological assessment (Modic)	PS is used for assessment of LBP^f^ (not for surgical outcome).
Schnee [43]	Retrospective	52 patients treated with PLIF and pedicle screw fixation for spondylolisthesis/mean follow-up 18.6 months	Efficacy of the technique measured as fusion rate and variation of PS scoring	Different version of PS (adaptation for patient)
Voorhies [13]	Prospective	110 patients operated for first decompression of lumbar root/mean follow-up 12 months	Identifying tools and risk factors to propose a predictive model of clinical success (6 measures set)	Analysis of prognostic factors and psychometric properties of PS. Statistical evidence of responsiveness to change
Woertgen [23]	Prospective	121 lumbar herniated disc patients/1 year follow-up	Different predictive factors of different scores (LBOS^g^, PS, pain grading scale)	Similar results on LBOS and PS, but no statistical analysis of psychometric properties

^a^Prolo Scale

^b^Posterior lumbar interbody fusion

^c^Visual analog scale

^d^Roland and Morris disability questionnaire

^e^Short-form 36

^f^Low back pain

^g^Low back outcome score

### Description of the Prolo Scale

The original scale is bidimensional. It is divided into an economic subscale (E) and a functional one (F), which present respectively the level of bearable work for the patient and the role pain plays in daily life. It consists of two 5-point Likert-type scales, where 1 is the worst condition and 5 is the best (Table 2).

**Table 2:** Economic and functional rating scale [1]

Economic status	
E_1_	Complete invalid
E_2_	No gainful occupation including ability to do housework or continue retirement activities
E_3_	Able to work but not at previous occupation
E_4_	Working at previous occupation part time or limited status
E_5_	Able to work at previous occupation with no restrictions of any kind
Functional status	
F_1_	Total incapacity (or worse than before operation)
F_2_	Mild-to-moderate level of low back pain and/or sciatica (or pain same as before operation but able to perform all daily tasks of living
F_3_	Low level of pain and able to perform all activities except sports
F_4_	No pain but patient has had one or more recurrences of low back pain or sciatica
F_5_	Complete recovery, no recurrent episodes of low back pain, able to perform all previous sport activities

The total score (ExFx) is obtained by adding scores of each subscale, resulting in a minimum score of 2 to a maximum of 10 points, which can be rated as excellent (10–9), good (8–7), fair (6–5) and poor (4–2). In the original study, Donald J. Prolo administered the scale to 34 patients who underwent posterior lumbar interbody fusion surgery.

Collected data were expressed as the ratio between the pre-surgery and final scores at 1-year follow-up. This ratio provided surgical outcome independent from surgical technique, and it was more objective than self-reported questionnaires (e.g., the Oswestry low back pain disability questionnaire—ODI) or anatomical examinations conducted by surgeons strictly related to the surgical success.

### The origin of the Prolo Scale

The original PS had been modified with respect to the one already used by Dawson, Urist and Lotysch in a retrospective study [4] conducted in 1981 on a sample of 58 patients who underwent intertransverse process lumbar arthrodesis from 1973 to 1979.

Similarly, Dawson and colleagues referred to a tool that had already been adopted long before, called the Massachusetts General Hospital Anatomic Economic Functional Rating System, which included three five-item subscales: anatomic, economic and functional (AEF) (Table 3) [5, 6].

**Table 3:** The Massachusetts General Hospital Anatomic Economic Functional Rating System [4]

A0	Pseudoarthrosis
A1	Unilateral pseudoarthrosis
A2	Insufficient unilateral fusion mass
A3	Contiguous fusion mass without hypertrophy
A4	Solid fusion with hypertrophy
E0	Complete invalid
E1	No gainful occupation
E2	Able to work but did not return to previous occupation
E3	Returned to previous occupation in part-time or limited status
E4	Returned to previous occupation without any restriction of any kind
F0	Pain worse than before surgery
F1	Level of LBP is the same as before operation but able to perform all daily tasks of living
F2	Low level of pain and able to perform all activities except sport
F3	No pain but patient has had one or more recurrences of LBP or sciatica
F4	Complete recovery, no recurrent episodes of LBP and able to perform all previous sport activities

Conversely to Dawson’s approach, Prolo and colleagues only considered items relative to economic and functional areas (EF), describing elsewhere the evaluation criteria of anatomical fusion, which was correlated with the scores obtained only by the surgeon. This choice could be explained by the small sample size or the authors’ intention to create a scale that is easy to administer and independent from the surgical technique.

Moreover, Prolo decided to modify the scoring method from the AEF system, with a minimum of 0 (disability) to a maximum of 4 points, to the EF system, with a minimum value of 1 (disability) to a maximum of 5 points.

### Diffusion of the Prolo Scale

Several researchers administered the original PS [7–34] as a main outcome or in association with other outcome measures, mostly in studies conducted on degenerative pathologies of the lumbar spine. Some authors used the PS by properly adapting items for the postoperative evaluation of function of other spinal districts, for example, the thoracic spine in case of fracture stabilization [35, 36] or discectomy [37] or the cervical spine.

In the early 1990s, some authors followed Prolo’s intention of creating a widely accepted assessment tool by publishing retrospective studies conducted on a significant population sample.

In 1992, Pappas et al. [7] carried out a retrospective study in which they administered the functional economic outcome rating scale to patients who underwent surgery with three different surgical procedures for lumbar hernia. Pappas and colleagues stated that the scale was a simple and useful tool for standard evaluation of the efficacy of different surgical techniques in opposition to self-report measures. They proposed that in future studies both the surgeon and the patient have to fill out the scale in order to allow a comparison between the results of the two different assessments. A discrepancy was found with respect to the stratification of combined scores. In fact, Prolo and colleagues proposed four outcome categories, excellent (10–9), good (8–7), fair (6–5) and poor (4–2), while Pappas organized results in only three categories: good (8–10 points), moderate (6–7 points) and poor (5 points or less). As a consequence, the threshold values were different for each class, and the cutoff value for poor outcome was different.

In 1994, Davis [8] administered the PS retrospectively and made use of direct evaluation, phone interviews and job agency databases. He examined long-term outcomes of different surgical procedures and compared his results to the study of Pappas. Davis highlighted the dearth of consensus on the meaning and quantification of long-term results, which varied between 4 and 20 years. He asserted that a follow-up longer than 4 years could be considered suitable to detect possible recurrences.

Similarly, retrospective studies were published years later: the purpose of the study of Schoeggl et al. [9] was to measure medium- and long-term surgical outcomes. The PS—as a self-reported questionnaire—was mailed to 672 patients who underwent microdiscectomy surgery between 1990 and 1998. The authors suggested further studies to compare results by making patients, surgeons and independent observers fill out the scale. After comparing their data and the results of other prospective studies, they suggested employing the PS as standardized criteria to evaluate postoperative surgery of the lumbar spine.

Since the end of the 1990s, debate has continued with regard to the most appropriate tool to measure the outcome and for data collection, and different comparison methods have been criticized. For instance, some authors doubted the accuracy and reliability of retrospective reports, in which, years after surgery, patients are asked to describe the difference between their own condition before and after the operation, overestimating surgical success [38, 39].

Other authors stated that it is necessary to make use of a multidimensional set of outcomes to evaluate complex pathologies like the ones affecting the lumbar spine. Among these, Deyo et al. [40] recommended a group of tests for the LBP, which was subsequently used by other authors [41].

In 2000, Berger et al. [10] criticized the indirect evaluation of phone interviews and questionnaires and published a study by using direct evaluation. The authors reported medium- and long-term outcomes (3–4 years) of 1,000 patients who had undergone lumbar surgery and had current work-related law suits. The authors examined subjects clinically with a direct evaluation and with the PS as the only semiquantitative measure of outcome. Data comparison showed a noticeable discrepancy between the low rate of neurological deficits and the considerable number of subjects unemployed because of chronic pain. The authors concluded that psychosocial factors had to be taken into account, and surgical efficacy could not be measured only by evaluating work-related conditions.

In 2002, Blount et al. [42] focused on elaborating standardized and multidimensional tools in order to reduce the risk of subjective bias as much as possible. The authors conducted a review of 27 studies on spinal fusion outcomes by finding the most common tools, and afterward they indicated a set of tools to measure the subsequent variables: general health status, lumbar disability, patient satisfaction, return to previous occupation, medication use and status of anatomical fusion. Especially, they suggested the “economic” version of Schnee [43] with respect to the return-to-work item, because it was the only available tool to quantify this area. In contrast, they did not recommend the Prolo Functional Scale to assess the spinal disability and preferred the ODI to evaluate lumbar outcomes and the Neck Disability Index to evaluate the cervical ones.

Furthermore, discrepancies between anatomical and functional outcomes are stressed by several authors. Porchet et al. [11] compared radiological findings and clinical examination by administering pain and disability scores. Concerning the PS, the correlation was not linear with respect to the others because of the difference between the group with severe disk conditions (sequestrum, extrusion) and the group with moderate disk conditions (bulging, protrusion). The author concluded that “poor” economic and functional levels constituted risk factors for severe disk pathology.

In other studies, controversial correlations were found between the radiological report and surgical success, depending on whether the outcome was obtained according to the patients’ perception or the surgeons’ criteria [42, 44]. Significant differences were reported between subjective satisfaction (67 %) and clinical success (39 %) [12].

In some cases, researchers chose integrated measures that included both the subjective perception of patients and the clinical ones of surgeons. Among these studies, Voorhies et al. [13] provided three definitions of clinical success related to the VAS, PS and surgeon examination, and Costa et al. [14] used a final cumulative score with the aim of assessing the efficacy of a lumbar fusion device by adding the VAS and PS scores.

Some randomized controlled trials (RCT) of high methodological quality used the PS as the primary outcome measure. In order to assess the efficacy of sequestrectomy as opposed to microdiscectomy, Thomé et al. [15] used the original PS along with the SF-36, VAS and patient satisfaction outcome. Dantas et al. [16] administered the scale to measure the results of two different stabilization techniques along with the Roland and Morris disability questionnaire (RMDQ) and ODI.

In several RCTs, the PS was considered an observational tool to measure post-surgical outcomes. Arts et al. [17] compared the efficacy of two surgical procedures, Peul et al. [18] compared early surgical intervention and prolonged conservative treatment for sciatica, Brox et al. [19, 20] evaluated the efficacy of lumbar fusion and conventional physical therapy vs. cognitive rehabilitation, and finally the recent RCT of Hellum et al. [21] examined the efficacy of a conservative protocol compared to disc replacement in patients with chronic LBP. Hence, in these studies and in many others, the PS was considered as a secondary outcome, whereas commonly the main ones were self-reported questionnaires that have been validated in several languages.

### Modified versions of the Prolo Scale

In 1997, the PS was modified by Schnee et al. [43], who administered a self-reported version of the scale to 52 patients who underwent lumbar fusion.

As reported in Table 4, non-relevant changes in the economic subscale were introduced so as to provide a more explicit correlation with daily activities, not necessarily work-related. The most evident change referred to the functional subscale instead, where items F3, 4 and 5 were simplified, and they emphasized the frequency and intensity of pain.

**Table 4:** The Prolo Economic and Functional Rating Scale (Schnee et al. [43])

Economic (activity) status		Functional (pain) status	
Grade	Description	Grade	Description
E1	Complete invalid (worse)	F1	Total incapacity (worse)
E2	No gainful occupation (including housework or retirement activities)	F2	Moderate-to-severe daily pain (no change)
E3	Working/active but not at premorbid level	F3	Low level of daily pain (improved)
E4	Working/active at previous level w/limitation	F4	Occasional or episodic pain
E5	Working/active at previous level w/o restrictions	F5	No pain

In particular, the original PS considered the score of the F3 item as low pain, which allows for daily activities but not sports, whereas the F4 item indicates absence of pain but recent recurrence of LBP (without any specification concerning the level of bearable activity). Absurdly, a patient with low pain and who is able to perform all activities except sports (E3F3 original scale) could get a lower score than a patient with recent recurrence who would not currently feel pain but is unable to perform certain activities (E3F4 original scale).

This modified version was named the “economic and functional rating scale” and was used by other authors [45–49] and recommended by Blount [42] for the economic subscale.

In 2000, Brantigan et al. [50] modified the scale in a multicenter-2-year retrospective randomized trial in which they administered a protocol that was created in the 1990s [51] and approved by the Food and Drug Administration (FDA) in 1999 in order to introduce a surgical device (I/F carbon cage) for posterior lumbar interbody fusion. The authors declined using common tools to assess the LBP (e.g., the ODI, RMDQ, etc.), yet they administered the PS because it was more useful to compare data from surgical studies carried out at different times. Nevertheless, they stated for the first time that the PS had not been validated yet; therefore, they suggested a modified version with 20 items (Table 5). This “modified Prolo Scale” presents, beyond the economic and functional subscales, which were different with respect to the original version, a pain subscale (P) and a medication subscale (M), both with five items. The authors affirmed that the PS already included outcomes of pain, function, economic status and use of pain medication, but in their study each of these parameters was evaluated separately. This difference influenced the final score, which could vary from a minimum of 4 to a maximum of 20 points. In their study, the authors of the modified Prolo Scale determined the clinical success at 2-year follow-up as excellent (20-17 points), good (16-13 points) and fair (12-9) with a minimal clinical importance difference (MCID) of 3 points. The evaluation was performed before and after surgery at 1-, 3-, 6-, 12- and 24-month follow-ups. The authors matched all criteria developed in 1997 by the FDA and considered pain relief, functional enhancement, and functional neuromuscular improvement as indexes of clinical success. These variables were measured by using both the new 20-point scale and the original 10-point scale. Because calculations of clinical success based on the 10-point Prolo Scale, the 20-point scale, and the FDA clinical success criteria did not differ statistically, results can be meaningfully compared to other studies using the Prolo score, including the clinical studies of different interbody fusion devices.

**Table 5:** Clinical evaluation scales—‘modified Prolo scale’ (Brantigan et al. [51])

Pain		Function		Economic		Medication	
P1	Excruciating or unbearable pain	F1	Total incapacity	E1	Unable to do tasks around the home	M1	10 or more hydrocodone tablets or equivalent
P2	Severe pain	F2	Able to do activities in the home	E2	Able to do tasks around the home but unable to work	M2	6–9 hydrocodone tablets or equivalent
P3	Moderate pain	F3	Able to do activities outside the home with limitation of moderate-demand activities	E3	Able to work at light or sedentary capacity	M3	3–5 hydrocodone tablets or equivalent
P4	Mild pain	F4	Limitation of strenuous activities or sports	E4	Able to work at moderate capacity	M4	Regular nonsteroidal anti-inflammatory drugs (NSAIDs) and/or occasional hydrocodone tablets
P5	No pain	F5	Able to do all activities	E5	Able to work at heavy capacity or previous occupation	M5	None or occasional NSAID or equivalent

Because of the sample size, the exact protocol definition and encouraging results, this study was taken as a reference system in the following years by several authors, who chose the modified version [52–58] or only some of its items. For instance, Weber [59] used the “Pain” subscale, Pellisé [39] the “Functional” and “Pain” subscales.

Since the study of Brantigan et al. [50] was carried out, three different versions of the PS have been administered to lumbar surgery patients: the original version, Schnee’s modified version and the 20-point one according to Brantigan et al. Another version of the scale, called the “modified Prolo scale,” was adapted for the cervical spine (Table 6). It was proposed by Davis in 1996 [60] to measure long-term outcomes after posterior decompression for cervical radiculopathy and was administered in a retrospective study.

**Table 6:** The Prolo Functional and Economic Outcome Rating Scale modified for postoperative cervical radiculopathy (Davis [60])

Score	Criteria
Economic status	
1	Complete invalid
2	No gainful occupation, including ability to do housework, school or retirement activities
3	Ability to work, but not at previous occupation: able to perform housework, school and retirement activities
4	Working at previous occupation part-time or with limited status
5	Able to work at previous occupation with no restrictions
Functional (social) status	
1	Total incapacity (worse than prior to operation)
2	Persistent neck and arm pain, persistent paresthesias, motor weakness same as prior to operation (able to perform tasks of daily living)
3	Moderate neck and arm pain, persistent paresthesias, minimal motor weakness
4	No neck or arm pain, persistent paresthesias in fingers, no motor weakness
5	No neck or arm pain, no paresthesias, no motor weakness, complete recovery, able to perform previous sports activities

The PS modified by Davis is mentioned in retrospective [61] and prospective studies [62] and RCTs [63, 64], and its use was recommended (with B strength) in the diagnosis and treatment of cervical radiculopathy “from degenerative disorders guidelines” (North American Spine Society, [65]).

Several studies we examined did not specify the exact version of the PS they adopted. As a consequence, researchers who did not know the whole evolution of the scale could have some difficulty understanding which version of this scale was used or might try to obtain that information from other parts of the article. Confusion increased when the authors described the scale they administered as “modified” although they had used the original version. Among these, Dreyzin and Esses [22] applied the evaluation system retrospectively to 20 patients treated for spondylolisthesis and spondylolysis with the aim of compared the efficacy of two different surgical procedures. The PS was administered only postoperatively by asking patients to evaluate surgical outcome. The authors probably only defined this version as the “modified Prolo Scale” because there were merely negligible differences in how to write the items (e.g., grade 1 vs. E1, etc.).

Conversely, other versions of the “modified Prolo Scale” were significantly different from the original one. For instance, Kuslich and colleagues [66] used a 6-point instead of a 5-point scale to assess lumbar pain. Furthermore, Kuslich used a thoroughly opposite rating system from Prolo: 1 point meant no pain and 6 points disabling pain, whereas Prolo considered 1 as poor outcome. The economic status was measured without providing any details on the load or activity type and only the percentage of patients that returned to work was reported.

Despite its differences from the original scale, Ohnmeiss and Guyer [67] mentioned the study of Kuslich in their review aiming to verify the most adequate follow-up time after surgery of spinal implant devices. In this study it was mentioned that Kuslich administered the “modified Prolo Scale” and Brantigan the “5-point Likert Scale for pain” instead.

### Psychometric properties of the Prolo Scale

In 1997, Woertgen et al. [23] administered the PS in a prospective study on 121 patients affected by lumbar hernia who underwent surgery, comparing this scale with another lumbar disability scale (the low back outcome score—LBOS). Four different instruments were administered: the LBOS, PS, pain grading scale and quality of life scale. The authors highlighted that data collected with the PS and LBOS were not statistically different; nevertheless, according to the scale in use, different prognostic factors could lead to different outcome measures. Some factors (postoperative duration of pain and duration of preoperative paresis) would affect the final outcome of all scales, while other factors would be specific only to one measure. In particular, according to the PS a positive SLR test before 30° and the ability to walk for 500 m would be predictive factors of poor outcome.

In 2002 Porchet et al. [11] conducted a cohort study on 394 patients with sciatica to verify the relationship between the clinical examination (measured on the RMDQ, SF-36, VAS and PS) and the radiological assessment according to Modic criteria. A significant inverse association (*P* < 0.001) was found between low levels of PS and high severity of disc disease, but the assumption of a linear correlation was rejected by statistical testing (*P* = 0.064). The authors reported that “having a poor functional status on PS (<5) represented a threefold risk of severe disc disease (OR = 2.91; 95 % confidence interval 1.74–4.87),” so the Prolo score was retained in the multivariate logistic model as an independent predictor of severe disc disease. In this study, the PS was used as a disability score and not as a tool to assess surgical outcome, as it was intended by the original researchers in 1986.

In 2007, Voorhies et al. [13] carried out a study that might be considered a validation study of PS. It was a non-randomized trial that investigated the surgical outcome of 110 sciatica patients by adopting a six-measure set (VAS, McGill Sensory/Affective Scores, Prolo Economic/Functional Scores, Modified Ransford Pain Drawing Score). The purpose of the study was to elaborate an outcome-predictive model to determine whether a score is able to predict clinical success. The authors took into account three ways to define “clinical success”: surgeon evaluation, 50 % or greater reduction in the VAS score, and combined PS score at the excellent level (8–10 points). The latter was reported as a 10-point version with little difference with respect to the original paper, but more understandable and easier to compile (Table 7).

**Table 7:** The modified Prolo economic and functional scores [13]

Prolo economic score (modified)	Prolo functional score (modified)
Complete invalid (confined to the home)	Severe pain (cannot do anything, somebody has to help you day to day)
No gainful occupation (including no housework and no retirement or leisure activities)	Moderate level of pain (able to take care of yourself without help, but can’t do anything else)
Able to work but not at your previous job (nor do the same types of housework or take part in all of your recreational activities or pastimes)	Low level of pain (able to do everything except sports, physically demanding leisure activities or heavy housework)
Working at previous job but on a part-time or light duty status (same kind of housework or retirement activities as before, but reduced in the amount of time and effort)	No pain now, but you have had one or more spells of pain recently
Able to work at previous job (or do other things) with no restrictions of any kind	Complete recovery, no pain, able to perform previous sport activities

The authors found statistically significant differences between pre- and postoperative data for all outcome measures (*P* < 0.001 for PS—see Table 8), confirming their sensitivity. Moreover, correlation between scores and comorbidity factors (preoperative pain, legal and psychiatric factors) was investigated, and it was shown that those factors strongly influenced the outcome prediction. However, the lack of indicators of reliability, repeatability and validity (criterion, content and construct) led us to conclude that PS has never been examined from the psychometrical point of view.

**Table 8:** Significance tests [13]: comparison of each outcome measure between pre- and postoperative status

Variable	*N*	Preoperative	Postoperative	*P* value
Prolo economic score	110	2.78 ± 1.24	3.65 ± 1.16	<0.001
Prolo functional score	110	2.04 ± 0.65	3.41 ± 1.02	<0.001
McGill sensory score	110	13.00 ± 7.42	5.56 ± 6.65	<0.001
McGill affective score	110	3.30 ± 3.70	1.53 ± 2.77	<0.001
Visual analog scale (VAS)	110	7.36 ± 1.94	3.21 ± 2.72	<0.001
Modified Ransford Pain Drawing Score [from Voorhies] (MRS)	110	1.30 ± 1.42	0.64 ± 1.25	<0.004

Nevertheless, some authors who referred to the existence of validation studies of the PS neither mentioned the study of Voorhies nor provided any references to support their statements.

As previously mentioned, in the study of Debusscher and Troussel [25] it was affirmed that the Prolo score modified by Dreyzin and Esses, VAS and ODI “are scientifically validated for assessment of LBP.” Furthermore, in 2010 Brotis et al. [34] stated that the PS had been standardized and validated in Greece, but only mentioned the studies of Blount [42] and Prolo [1]. Finally, in 2007 Alrawi and colleagues [62] used the Davis modified version to examine the surgical outcome of cervical radiculopathy, and they stated that clinical evaluation was carried out by means of a validated scoring systems (the Prolo functional and economic system).

## Discussion

To date, there is insufficient consensus about the most adequate and reliable tool to measure lumbar surgical outcomes, and this prevents the comparison of the results among different clinical studies. In order to investigate such a complex condition as lumbar pathology, there is large consensus among authors as to the need to adopt a multidimensional set of measures that also allows considering comorbidity factors and reduces subjective bias.

The PS has been adopted for several years because it is easy to administer and useful for comparing a significant amount of data from surgical studies carried out at different times. Even though Voorhies [13] and Woertgen [23] demonstrated the scale sensitivity among a battery of tests, no thorough validation study was found in the current literature.

The original ten-point scale is widely used; however, the presence of two modified versions [43, 50] and the unclear indications given by authors can easily lead to mistakes by those who do not thoroughly know the evolution of the scale. Hence, in future studies, we strongly suggest specifying the version in use. In recent studies, PS has usually been considered a secondary outcome, whereas the primary measures consisted of validated specific tools based on patient perception (the ODI, RMDQ, SF-36).

Nonetheless, among the studies that used a validated scoring system, there is a lack of consensus about what clinical success means, as the study of Tafazal and Sell showed [68]. The authors stated that the outcome measured by means of three different scales (the ODI, LBOS, VAS), in order to achieve a good or excellent outcome, varies depending on the surgical procedure. In fact, data confirmed that the minimum clinically important difference (MCID) obtained for discectomy surgery is higher than the one for decompression or fusion surgery. This article shows that a single scoring method to assess postoperative outcome could be considered insufficient regardless of surgical technique.

In the current literature, the presence of new multidimensional tools such as the Core Outcome Measures Index [69, 70] to assess the LBP and the minimum core outcome set [71] for lumbar surgical outcome leads us to state that the issue concerning the lack of homogeneity in outcome measures still exists.

We suggest that future studies specify the exact version of the scale they used and thoroughly investigate the psychometric properties (reliability, validity and responsiveness) of questionnaires employed to evaluate the results of spinal surgery.
